# Unmet Rehabilitation Needs after Traumatic Brain Injury across Europe: Results from the CENTER-TBI Study

**DOI:** 10.3390/jcm10051035

**Published:** 2021-03-03

**Authors:** Nada Andelic, Cecilie Røe, Olli Tenovuo, Philippe Azouvi, Helen Dawes, Marek Majdan, Jukka Ranta, Emilie I. Howe, Eveline J.A. Wiegers, Cathrine Tverdal, Ida Borgen, Marit V. Forslund, Ingerid Kleffelgaard, Hilde M. Dahl, Louis Jacob, Mélanie Cogné, Juan Lu, Nicole von Steinbuechel, Marina Zeldovich, CENTER-TBI Participants and Investigators

**Affiliations:** 1 Department of Physical Medicine and Rehabilitation, Oslo University Hospital, 0424 Oslo, Norway; CEROEE@ous-hf.no (C.R.); emihow@ous-hf.no (E.I.H.); idmbor@ous-hf.no (I.B.); mavfor@ous-hf.no (M.V.F.); UXINFF@ous-hf.no (I.K.); 2 Research Centre for Habilitation and Rehabilitation Models and Services (CHARM), Faculty of Medicine, Institute of Health and Society, University of Oslo, 0373 Oslo, Norway; 3 Faculty of Medicine, Institute of Clinical Medicine, University of Oslo, 0318 Oslo, Norway; UXTVEC@ous-hf.no (C.T.); hilde@legesiden.no (H.M.D.); 4 Turku Brain Injury Centre, Neurocenter, Turku University Hospital, and Clinical Neurosciences, University of Turku, 20500 Turku, Finland; olli.tenovuo@pp.inet.fi; 5 AP-HP, GH Paris-Saclay, Hospital Raymond Poincaré, 92380 Garches, France; philippe.azouvi@aphp.fr; 6 Université Paris-Saclay, UVSQ, Inserm, CESP, UMR 1018, Team DevPsy, 94807 Villejuif, France; 7 Centre for Movement, Occupational and Rehabilitation Sciences, Oxford Institute of Nursing, Midwifery and Allied Health Research, Oxford Brookes University Oxford, Oxford OX3 0BP, UK; hdawes@brookes.ac.uk; 8 Oxford Health Biomedical Research Centre, Oxford Health NHS Foundation Trust, Oxford OX3 7JX, UK; 9 Department of Public Health, Institute for Global Health and Epidemiology, Faculty of Health Sciences and Social Work, Trnava University, 91843 Trnava, Slovakia; mmajdan@truni.sk; 10 VTT Technical Research Centre of Finland Ltd., FI-02044 Espoo, Finland; Jukka.Ranta@vtt.fi; 11 Department of Public Health, Erasmus MC, University Medical Center, 3000 CA Rotterdam, The Netherlands; e.wiegers@erasmusmc.nl; 12 Department of Neurosurgery, Oslo University Hospital, 0424 Oslo, Norway; 13 Department of Psychology, Faculty of Social Sciences, University of Oslo, 0424 Oslo, Norway; 14 Department of Child Neurology, Oslo University Hospital, 0424 Oslo, Norway; 15 Faculty of Medicine, University of Versailles Saint-Quentin-en-Yvelines, Montigny-le-Bretonneux, 78035 Versailles, France; louis.jacob.contacts@gmail.com; 16 Research and Development Unit, Parc Sanitari Sant Joan de Déu, CIBERSAM, Universitat de Barcelona, 08830 Barcelona, Spain; 17 Physical and Réhabilitation Médecine Department, University Hospital of Rennes, 2, rue Henri-le-Guilloux, 35000 Rennes, France; melaniecogne@hotmail.fr; 18 Department of Family Medicine and Population Health, Virginia Commonwealth, Division of Epidemiology, University of Richmond, Richmond, VA 23173, USA; juan.lu@vcuhealth.org; 19 Institute of Medical Psychology and Medical Sociology, University Medical Center Göttingen, 37073 Göttingen, Germany; nvsteinbuechel@med.uni-goettingen.de

**Keywords:** rehabilitation needs, traumatic brain injury, disability, outcome

## Abstract

This study aims to assess rehabilitation needs and provision of rehabilitation services for individuals with moderate-to-severe disability and investigate factors influencing the probability of receiving rehabilitation within six months after traumatic brain injury (TBI). Overall, the analyses included 1206 individuals enrolled in the CENTER-TBI study with severe-to-moderate disability. Impairments in five outcome domains (daily life activities, physical, cognition, speech/language, and psychological) and the use of respective rehabilitation services (occupational therapy, physiotherapy, cognitive and speech therapies, and psychological counselling) were recorded. Sociodemographic and injury-related factors were used to investigate the probability of receiving rehabilitation. Physiotherapy was the most frequently provided rehabilitation service, followed by speech and occupational therapy. Psychological counselling was the least frequently accessed service. The probability of receiving a rehabilitative intervention increased for individuals with greater brain injury severity (odds ratio (OR) 1.75, CI 95%: 1.27–2.42), physical (OR 1.92, CI 95%: 1.21–3.05) and cognitive problems (OR 4.00, CI 95%: 2.34–6.83) but decreased for individuals reporting psychological problems (OR 0.57, CI 95%: 1.21–3.05). The study results emphasize the need for more extensive prescription of rehabilitation services for individuals with disability. Moreover, targeted rehabilitation programs, which aim to improve outcomes, should specifically involve psychological services to meet the needs of individuals recovering from TBI.

## 1. Introduction

Physical, cognitive and emotional problems after traumatic brain injury (TBI) may cause physical, behavioral and psychosocial impairments, work disability [1], and an overall reduction in health-related quality of life [2]. These problems require a wide range of rehabilitation services delivered to both inpatients and outpatients after TBI by medical, allied health and social care professionals. Such services include nursing services, physical therapy, occupational therapy, psychological and neuropsychological services, speech therapy, social worker aid, vocational services, and more [3].

TBI rehabilitation is beneficial in that it improves patients’ functional outcomes beyond those expected from spontaneous recovery [4,5]. The key criteria for successful health and rehabilitation services are the inclusion of, and access to, well-coordinated multidisciplinary processes, which address the varying needs of patients with TBI [5,6]. Despite this, existing studies of rehabilitation needs and the use of rehabilitation services following TBI indicate a lack of multidisciplinary rehabilitation services during acute care [7,8]. Furthermore, traditional rehabilitation services, such as physical, occupational, and speech and language therapies are far more likely to be used than psychological counselling during the first three months post-TBI [3] or during the post-acute care after severe TBI [9]. As a result, more than one-third of patients may present unmet rehabilitation needs in the long-run following TBI [10]. These unmet needs may be attributable to the prevailing community-level approach where rehabilitation consists of interventions focusing on physical functioning, rather than a systematic multidisciplinary approach based on individual needs [11]. A lack of resources allocated for rehabilitation of variable post-injury needs likely also contributes.

Service utilization may vary based on demand and specific needs across different demographics and injury severity characteristics, and service provision and access may further vary across regions and countries. Studies determining the predictive value of socio-demographics on the use of rehabilitation services after TBI show contradictory results. Some report sex differences in service utilization, such as higher healthcare use among females [7,10]. In other studies, however, males received more services [12], or no correlation between sex and service utilization was observed [3,9]. Furthermore, some studies report that younger individuals more often received rehabilitation after TBI [10,12,13], while others report that older individuals more often received such services [14,15]. More frequent service utilization has been reported to be more frequent among patients after severe TBI [7,16,17], among those with more severe overall trauma [7] and in those with more severe disabilities [16]. Other studies report that unmet rehabilitation needs were significantly higher in individuals with a less severe disability outcome [10].

Johnstone et al., report that geographical regions might influence access to TBI rehabilitation services [18], which may explain the presence of contradictory study results. Supporting this, a survey study by Cnossen et al. found substantial variation in the general policies governing the structure and process of acute in-hospital rehabilitation and referrals to post-acute rehabilitation among the European neurotrauma centers [13]. However, most studies on rehabilitation needs and the use of rehabilitation services are conducted within the United States (USA) [19], and few occur outside the USA [7,9,20]. Therefore, to improve the service provision, a better understanding of rehabilitation needs and culture- and country-related differences is required. To gain this understanding, additional studies must be conducted in a diverse range of countries. 

To this end, the Collaborative European NeuroTrauma Effectiveness Research in TBI (CENTER-TBI) [21] observational study was conducted from 2014 to 2017 in Europe and Israel. A recently published paper from this large multicenter study including all injury severity levels reported the prevalence of rehabilitation use to be 32.4% in the first year after TBI across the European countries and identified predictors of access to rehabilitation following TBI [7]. However, an assessment of the specific rehabilitation needs and service provision among sub-group of individuals with functional impairments and moderate-to-severe disability outcomes according to the Glasgow Outcome Scale—Extended (GOSE) [22] has not yet been performed. This subgroup was chosen as this impairment level is considered to generate needs for rehabilitation. Such a study is essential for further development of best practices for TBI rehabilitation service provision and delivery in Europe. 

The aims of this study were as follows:

1. To assess the rehabilitation needs and provision of rehabilitation services for individuals exhibiting TBI-related impairments and disability across Europe during the first six months post-injury.
2. To investigate whether sociodemographic, premorbid, and injury-related factors predict the probability of receiving rehabilitation services at three and six months after injury.

Based on previous studies, we hypothesized that:

1. There will be a high percentage of rehabilitation needs among individuals with TBI-related impairments and disabilities in the first six months following TBI.
2. There will be an association between the probability of receiving rehabilitation and age, sex, injury severity, comorbidities, and geographical regions.

## 2. Methods

### 2.1. Study Design

Participants were recruited from the CENTER-TBI project, a multicenter, prospective observational longitudinal cohort study that aims to better characterize TBI and to identify the most effective interventions for managing TBI [21]. The core study enrolled patients with all TBI severities from 18 countries (Austria, Belgium, Denmark, France, Finland, Germany, Hungary, Israel, Italy, Romania, Serbia, Spain, Sweden, Latvia, Lithuania, Norway, the Netherlands, and the United Kingdom) and 65 participating centers between 19 December 2014 and 17 December 2017. Inclusion criteria were a clinical diagnosis of TBI, an indication for a computed tomography (CT) scan and presentation to a medical center within 24 h of injury. Individuals were excluded if they had a severe pre-existing neurological disorder that could bias outcome assessments. Three strata were used to prospectively differentiate patients by clinical care pathway: emergency room (ER; patients evaluated in the ER and discharged afterwards), admission (patients admitted to a hospital ward), and intensive care unit (ICU; patients who were initially admitted to the ICU). The main descriptive findings of CENTER-TBI have been published elsewhere [23].

This study is a part of Work Package 14 (WP-14), aimed at describing optimal ways to provide different levels of care to individuals after TBI. The complex issue of rehabilitation is a major focus of this WP. Thus, the assessments included in the current study were from the original CENTER-TBI study.

### 2.2. Participants

**Figure 1:**
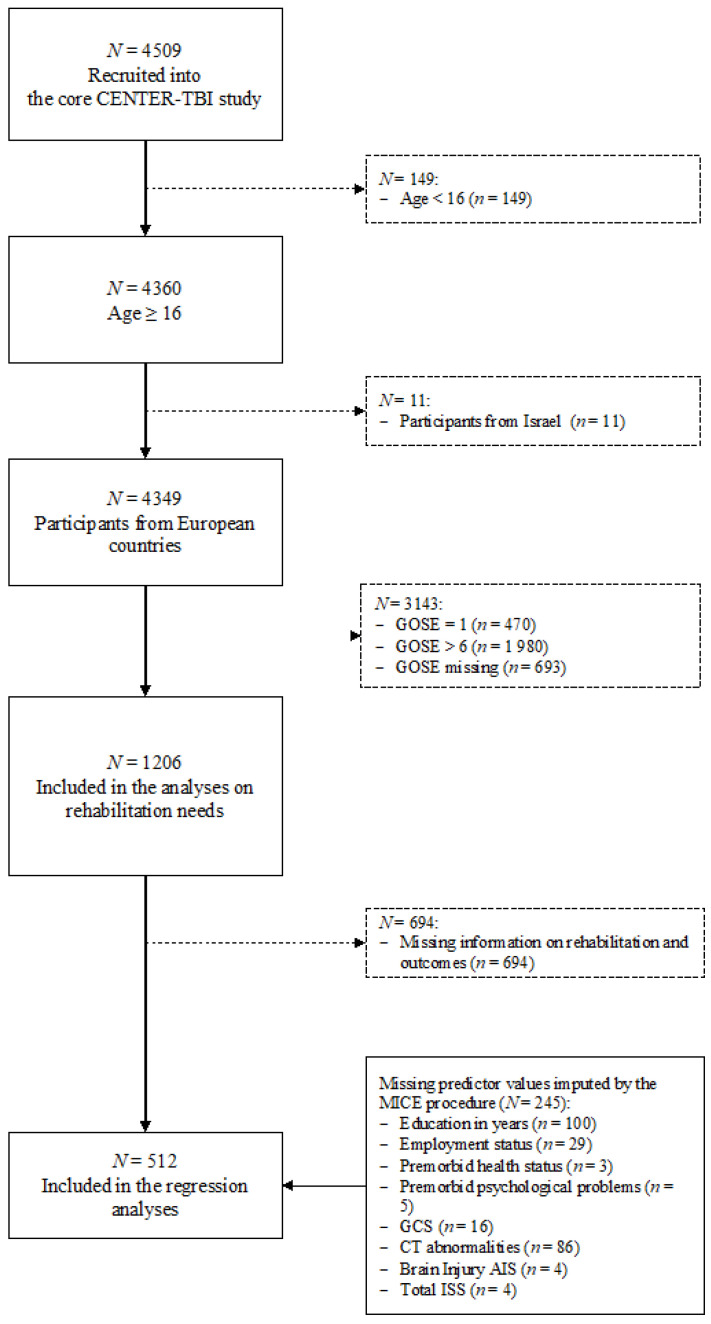
Sample attrition flowchart.

To assess rehabilitation needs, 1206 individuals over 16 years of age were selected from the 17 European countries involved in the CENTER-TBI project. Participants from Israel (*n* = 11) were excluded as this study assesses the rehabilitation needs across the Europe. All selected individuals showed TBI-related moderate-to-severe disability outcome—as measured by the GOSE score—at their six-month follow-up appointment. The GOSE score consists of an eight-point scale (1: dead, 2: vegetative state, 3/4: lower/upper severe disability, 5/6: lower/upper moderate disability and 7/8: lower/upper good recovery) and is based on structured clinical interviews or self- or proxy- ratings. Besides the GOSE, the patient- or proxy-reported version of the GOSE questionnaire (GOSE-Q) [24] administered by postal mail was used to collect information on disability outcomes. The GOSE-Q score was centrally computed, combined with the GOSE interview score and integrated into the CENTER-TBI database. As the questionnaire version does not distinguish between vegetative state and lower severe disability categories, the two were collapsed into one group. The present study includes individuals exhibiting lower-severe to upper- moderate disability outcomes six-months post-injury. For the regression analyses, only those individuals who provided information on received rehabilitation services and impaired outcomes were selected resulting in an effective sample size of 512 individuals. For more details on samples attrition, see Figure 1. 

### 2.3. Instruments

#### 2.3.1. Sociodemographic, Premorbid, and Injury-Related Data

Sociodemographic and injury-related data were collected at the study’s inception. Data included sex (female or male), age in years, years of education, living situation (alone or not alone) and work participation (employed, unemployed, and other—i.e., retired, studying, or homemaker). 

The geographical region was determined by the country of the participating sites. Based on the EU Vocabularies (EuroVoc) classification [25], countries were stratified into Western (Austria, Belgium, France, Germany, The Netherlands and the United Kingdom); Southern (Italy and Spain); Northern (Denmark, Finland, Latvia, Lithuania, Norway and Sweden); and Central and Eastern Europe (Hungary, Romania, and Serbia). Due to the small number of participants, Southern and Eastern European regions were collapsed into one group.

Premorbid somatic health status was assessed according to the classification of the American Society of Anesthesiologists Physical Status Classification System [26]. This classification originally comprised four groups: healthy, mild systemic disease, severe systemic disease and severe systemic disease with constant threat to life. The latter two were collapsed into one category (termed severe systematic disease) due to a low number of cases. 

Injury-related information covered: (i) the injury mechanism (e.g., road traffic accident, falls, other); (ii) clinical care pathways (ER, ward, and ICU); (iii) TBI severity as measured by the Glasgow Coma Scale (GCS [27] within the first 24 h after injury as both a continuous variable and classified into mild (13–15), moderate (9–12), and severe (3–8); (iv) the presence of intracranial injuries on the first CT scan (absent/present); (v) the Abbreviated Injury Scale [28] (AIS; Brain injury AIS, score ≥ 3 considered as severe intracranial injury), and (vi) overall injury severity as measured by the Injury Severity Score (ISS) [29]. The ISS ranges from 0 (no trauma) to 75 (not survivable) and a score > 15 is considered major overall trauma [30]. 

#### 2.3.2. Assessment of Rehabilitation Needs

For the assessment of rehabilitations needs, the following five outcome domains were identified: problems with activities of daily life, physical, cognitive, speech and psychological problems.

*Problems with activities of daily life* were measured using the scale Daily Life and Autonomy of the Quality of Life after Brain Injury Scale (QOLIBRI) [31,32] with a cut-off score of 60 and lower scores indicating impairment. 

*Physical problems* covering mobility (e.g., problems with mobility or walking) and movement ability (e.g., problems with movement of the hands or arms) were self-reported and assessed by two questions from the Participant Questionnaire of the CENTER-TBI project. Individuals who responded affirmatively to at least one yes/no question were considered impaired. 

*Problems with cognition* were measured using three questions from the Rivermead Post-Concussion Symptoms Questionnaire (RPQ) [33]. ‘Forgetfulness, poor memory’, ‘poor concentration’ or ‘taking longer to think’ were rated at least as mild ≥2 [34]. Individuals who reported at least one of these symptoms were considered impaired. 

*Problems with speech and language* were measured using the yes/no question ‘problems with speaking or understanding others’ in the Participant Questionnaire mentioned above. 

*Psychological problems* were considered to be present if at least one of the following outcomes was rated as impaired:

(i) *Post-traumatic stress disorder (PTSD)* was captured by the Posttraumatic Stress Disorder Checklist-5 (PCL-5) [35]. The PCL-5 measures 20 symptoms of PTSD based on the Diagnostic and Statistical Manual of Mental Disorders, 5th edition (DSM-5) [36] using a five-point Likert scale (from 0, ‘not at all’, to 4, ‘extreme’). The total score ranges from 0 to 80, with higher values indicating greater impairment. A cut-off value of 33 was applied to determine clinically relevant PTSD [37].
(ii) *Depression* was assessed with the Patient Health Questionnaire (PHQ-9) [38]. The PHQ-9 assesses symptoms of depression using nine items and a four-point Likert scale (from 0 ‘not at all’ to 3 ‘nearly every day’). The PHQ-9 total score ranges from 0 to 27, with higher values indicating greater impairment. A cut-off value of 10 was applied to determine clinically relevant depression [38,39].
(iii) *Anxiety* was assessed with the self-reported Generalized Anxiety Disorder seven-item scale (GAD-7) [40]. The GAD-7 uses seven items and a four-point Likert scale (from 0 ‘not at all’ to 3 ‘nearly every day’). The total score ranges from 0 to 21. Higher values indicate greater impairment, with a cut-off value of 10 indicating impairment [40].

#### 2.3.3. Professional Help and Rehabilitation Services

Information concerning professional help and rehabilitation services provided after TBI was based on self-report. Participants were asked to report any specialized professional help they received because of the TBI, including help for problems with speaking (e.g., speech therapy), memory or attention (e.g., cognitive rehabilitation), problems with movements (e.g., physiotherapy), help for problems with looking after themselves (e.g., occupational therapy), emotional difficulties, behavioral regulation and fatigue (e.g., psychological services). Multiple answers were allowed. Participants were then asked to utilize the following response categories to describe the rehabilitation they received because of the TBI with the following response categories: inpatient/residential rehabilitation, out-patient/community rehabilitation, or no rehabilitation. This information contained no reference to the impaired outcome domains. 

### 2.4. Statistical Analyses

#### 2.4.1. Unmet Rehabilitation Needs

First, we identified rehabilitation needs at three and six months after TBI using the methods and instruments described above. The amount of professional help and rehabilitation services provided was measured using the ratio of total impaired individuals versus individuals who had received professional or rehabilitation services. Individuals with impairments who did not receive inpatient/residential or outpatient/community rehabilitation services were considered to have unmet rehabilitation needs.

To provide an overview of these unmet rehabilitation needs, we classified the ratio into three groups based on the following the cut-off values: covered needs (75% or more of the impaired individuals receiving respective services), semi-covered needs (less than 75% but more than 25% of impaired individuals receiving respective services), and uncovered needs (less than 25% receiving respective services). Coverage of rehabilitation needs is provided per outcome category. For example, if a person reported problems with activities of daily living and psychological problems but received only occupational rehabilitation and no psychological treatment, his or her needs were covered to 100% in the “activities of daily life” outcome category and not covered in the “psychological problems” category. If each person who reported an impairment in a category received appropriate rehabilitation, the total coverage was 100%. Based on the results of this classification, the coverage of rehabilitation needs was subsequently ranked according to the five outcome domains at three and six months after TBI, respectively.

Differences between timepoints were examined using chi-square tests with Bonferroni-corrected *p*-values for multiple outcome comparisons (e.g., $\alpha_{adj}$ = 0.05/5 = 0.01 for the five outcome domains or $\alpha_{adj}$ = 0.05/4 = 0.0125 for the four recovery status groups).

#### 2.4.2. Prediction of Probability of Receiving Rehabilitation

Logistic regression was applied separately to predict the probability of receiving rehabilitation at three and six months post-injury adjusting for factors suggested by previous research. The dependent variable represents provided rehabilitation services (0 = no rehabilitation vs. 1 = inpatient or outpatient rehabilitation). Nineteen independent variables include sociodemographic information at baseline (sex, age, living situation, years of education, employment status, and geographical region); premorbid health status (overall health and mental health); injury-related factors such as injury cause, clinical care pathways, TBI severity (GCS, abnormalities on the first CT scan, brain injury) and overall injury severity. In addition, impairments in the five outcome domains were included. 

The following steps were performed to run the regression analyses. First, the missing values in the predictor variables (varying from 1% for the premorbid health status to 19% for the number of education years) were imputed using multivariate imputation by the chained equations (MICE) procedure [41]. The MICE procedure is a multi-step approach that is able to account for the influence of multiple factors simultaneously and impute missing values based on variable type (e.g., continuous, dichotomous, or polytomous) [42]. Missing values are imputed by applying a series of corresponding regression models multiple times and using pooled data for target analyses to avoid bias [41]. Second, the initially defined model was estimated. Factors contributing to the probability of receiving rehabilitation were chosen according to the backward stepwise Akaike’s information criterion (AIC) procedure with a liberal *p*-value of 0.157 corresponding to a predictor with 1 degree of freedom. While the regression model was defined based on previous research, a liberal *p*-value was used to justify variable selection for the final model reported [43]. A bootstrapping validation procedure assessed the goodness of fit on the imputed data providing optimism-corrected fit indices. Nagelkerke’s R^2^ [44] was used to assess whether the estimated model differed from a hypothetical null-model without any predictors. The Nagelkerke’s R^2^ ranges from 0 to 1 with higher values indicating the relative improvement of the estimated model compared to a null model. The area under the receiver operating (ROC) curve (AUC), which corresponds to the c-statistic [45,46], was used to assess model’s ability to distinguish between individuals who received rehabilitation services and those who did not. AUC values from 0.70 to 0.80 were considered acceptable, while values over 0.80 were considered excellent and over 0.90 were considered outstanding [47]. 

All analyses were performed with the R version 4.0.2 [48] using the packages ‘mice’ [49] for the missing values imputation and ‘psfmi’ [50] for the model estimation, selection of potential predictors, and bootstrapping validation. The alpha level was set at 0.05 except for multiple comparisons between three and six month assessments and the stepwise AIC procedure.

## 3. Results

### 3.1. Sample Characteristics

The study sample (*N* = 1206) consisted of majority males (67.7%) and had a mean age of 49.3 (*SD* = 18.9 years, *Mdn* = 50, *IQR* = 77). Half of the participants had at least 13 years of education and were employed (52.5%) at the time of the injury, and half were residents of Western European countries (49.2%). Most individuals (52.4%) reported no somatic health problems and no psychological problems (80.3%) prior to TBI. Road traffic accidents (45.6%) were the most common cause of TBI, and most of patients with TBI were admitted to the ICU (71.2%). Of the sample group, 49.1% sustained a mild TBI, 11.7% sustained a moderate and 35.2% a severe TBI. Finally, 62.2% showed abnormalities on the first CT-scan (Table 1). 

### 3.2. Rehabilitation Needs

Almost all participants had at least one impaired outcome at both three and six months post-TBI (91% and 89%, respectively). Over 40% reported suffering from at least two or three impairments, and nearly 9% were impaired in all five areas (problems with activities of daily life and physical, cognition, speech, and language and psychological problems) at three and six months after TBI. 

Table 2 summarizes the distribution of N = 1206 individuals with GOSE scores from 2/3 to 6 and outcome domains at three and six months after TBI, respectively. At three months after TBI, participants mostly exhibited difficulties with physical problems closely followed by cognitive problems. At six months, cognitive problems were more frequently reported than physical problems (with approximately half of the participants reporting cognitive problems). Other outcome areas were nearly equally distributed at three and six months after TBI with speech and language problems showing the lowest frequency of impairments. Across all time points and all outcome domains, individuals admitted to the ICU exhibited the highest degree of impairment. 

### 3.3. Coverage of Rehabilitation Needs

Table 3 presents information on the type of rehabilitation services provided in the defined outcome domains. At three and six months, physiotherapy was the most frequently prescribed service (66% and 71%, respectively) followed by speech (39% and 43%, respectively) and occupational therapy (37% and 43%, respectively). Approximately 33% of patients with cognitive problems at three months and 37% at six months self-reported that they received cognitive rehabilitation. Psychological services, including professional help with anxiety, depression, stress, behavioral problems and fatigue, were provided to approximately 26% and 30% of the individuals who reported psychological problems at three- and six-months assessments, respectively.

Irrespective of the outcome area impaired, individuals admitted to the ICU participated more frequently in in-patient rehabilitation programs at both time points. In contrast, out-patient treatments were used by those admitted to the ER and then discharged, followed by individuals treated in the hospital ward (see Table A1 in Appendix A). 

**Figure 2:**
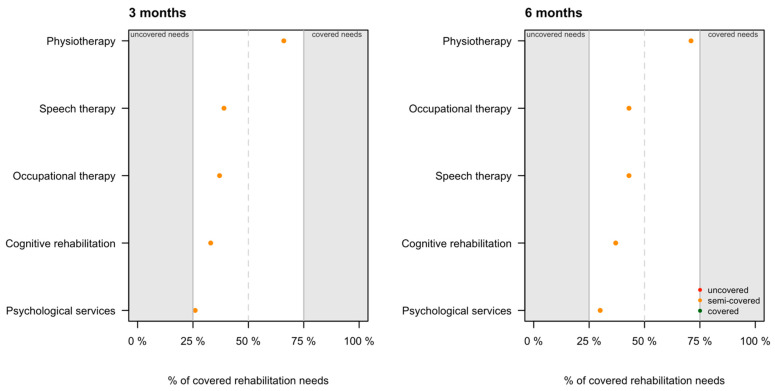
Rehabilitation services provided to individuals with the respective impaired outcomes. Services were ranked based the relative frequency (e.g., physiotherapy was provided more frequently than speech therapy at three months and more frequently than occupational therapy at six months after TBI).

Overall, at both time points rehabilitation needs were semi-covered across all outcome domains (i.e., between 25% and 75% of those impaired received the necessary support)—the only ranking difference between the time points involved occupational and speech therapies. While occupational therapy was a more frequent part of the rehabilitation program component at three months post-TBI, speech therapy was more highly ranked at six months. At each time point, psychological services were the least frequent service provided relative to documented impairment. For details, see Figure 2.

Of the 1206 participants, approximately 30% received inpatient rehabilitation at three and six months after TBI, irrespective of the impaired outcome domains. Individuals admitted to the ICU displayed the highest rate of involvement in rehabilitation programs (35% and 39% at three and six months, respectively). At three months post-TBI, 13% of individuals admitted to a hospital ward participated in inpatient rehabilitation programs, whereas at six months after TBI, only 8% received inpatient treatment. Among all groups, individuals admitted to the ER and subsequently discharged showed the lowest percentage of participation in rehabilitation programs with 2% and 5% at three and six months, respectively. Overall, about 15% of participants received outpatient rehabilitation regardless of the impaired outcome domains. Individuals admitted to the ER and then discharged (17% and 20% at three and six months, respectively) were closely followed by those admitted to a hospital ward (14% and 18%) in terms of the frequency with which they received outpatient rehabilitation services. Approximately 12% and 14% of those admitted to the ICU took part in outpatient rehabilitation programs at three and six months, respectively. Around one-fourth did not take part in any rehabilitation programs. Nearly one-third provided no information on rehabilitation treatments at each time point with individuals admitted to the ICU showing the highest number of missing values (37% and at 34% at three and six months, respectively). For details, see Table A1 in Appendix A.

The group of individuals with low recovery status (GOSE scores of 2/3) had the highest number of missing values (i.e., no information regarding rehabilitation was available). Still, the lower the GOSE score, the lower was the likelihood of outpatient rehabilitation. For further details, see Table 4. 

Table 5 presents information regarding rehabilitation type (no rehabilitation, in- or outpatient services) provided for patients with impaired outcomes. The data includes no information about the primary reason for the rehabilitation (e.g., mobility issues, cognitive and communication problems, or both). 

### 3.4. Prediction of the Probability of Receiving Rehabilitation

Since there was almost no difference in the three- and six-months prediction results, only the six months results are reported here. 

At six months post-TBI, a more severe TBI (i.e., lower GCS and higher Brain Injury AIS), the presence of physical and cognitive problems, and the absence of psychological problems contributed significantly to a higher probability of receiving rehabilitation services. The total ISS and clinical pathways (ER, ward, and ICU) were included in the model according to the stepwise AIC procedure, but they generated no significant results. The corrected AUC/c-statistic produced the value of 0.84 (optimism correction of −0.01), indicating a strong model with excellent discriminating ability [47]. According to the model, individuals who had received rehabilitation had a higher predicted probability (i.e., 84%) to be identified as such than individuals who had not received rehabilitation. The corrected pseudo R2 = 0.42 (optimism correction of −0.01) indicated prediction improvement from the null model to the final model. For more details, see Table 6.

## 4. Discussion

The present study is, to our knowledge, the first study to assess rehabilitation needs and the use of rehabilitation services in individuals with moderate-to-severe disability after TBI from the European perspective. 

We chose to focus on the more severe spectrum of the TBI population as their rehabilitation needs are unequivocal [51]. The male predominance is in accordance with the epidemiologic characteristics of this population [52], and the median age of 50 years is representative for the CENTER-TBI population [23]. Close to half of the present population had pre-existing somatic conditions and 10% of these had more severe somatic problems. In the CENTER-TBI context, the GOSE score assesses the overall functional problems in contrast to the original use of GOSE which evaluated only TBI-related disability outcomes [53]. Distinguishing the TBI-related disability from the overall disability may be a challenge, and the present analyses stress the different impairments in functioning. 

Consistent with our first hypothesis, rehabilitation needs were reported by 90% of individuals, and these often presented within several outcome domains. However, the needs were only semi-covered across all outcome areas. Cognitive impairments were most common in the present study, followed by physical problems, problems with daily life activities, and psychological problems and speech problems. The burden of cognitive problems in the TBI population is well known [2], yet provision of physiotherapy continues to dominate. In the present study, physiotherapy was delivered to nearly half of the patients who did not report physical problems, which raises concerns regarding the appropriate allocation of rehabilitation resources. Our finding might be related to the fact that physiotherapy services are the ones most available across countries. In addition, the health payer system (a health care system, insurance companies and similar), may offers easy-access and full reimbursement for this service [9]. However, a detailed description of physical functioning and impairments beyond coarser mobility impairments was not included in this study, and these impairments could require physiotherapy. 

In contrast, psychological services were provided to less than one-third of the individuals reporting psychological problems, representing the service with the lowest coverage of existing needs. Psychological problems may develop for various reasons, including as a result of the brain injury, secondary to other problems in functioning or because of unmet health care and rehabilitation needs related to the TBI, such as a lack of meaningful activities at an appropriate level or problems coping with injury consequences [10]. According to Jennekens et al.’s systematic review [19], a ‘prerequisite for offering adequate help is that health care providers understand the needs of these patients.’ The present results clearly underscore an urgent need for a stronger focus on monitoring psychological domains during TBI rehabilitation and the adjustment of rehabilitation service delivery to meet the needs of the TBI population. 

Inpatient rehabilitation services were provided to 30% of the present population, and unsurprisingly, the highest coverage was extended to individuals admitted to the ICU, who are assumed to have the most severe injuries. Rehabilitation is considered highly beneficial for individuals after TBI; however, only 15% of patients received outpatient rehabilitation, which is considerably low given the long-term rehabilitation needs in the TBI population evidenced in earlier studies [10,54].

The applied model showed a good predictive ability and indicated that the probability of receiving rehabilitation depends primarily on injury-related factors, such as brain injury severity and impaired outcome domains. Consistent with our second hypothesis, injury severity, as well as physical and cognitive problems did increase the probability of receiving rehabilitation services, yet psychological problems decreased this probability. It is particularly worrisome that patients with psychological problems—assumed to be a vulnerable population—receive insufficient health care services [20]. 

Contrary to our second hypothesis, age, sex, and geographical regions (North, West and Southern/Eastern Europe) did not predict the probability of receiving rehabilitation. One possible explanation for this finding is an increased focus on the equality of health care during the last decade. In particular, the provision of rehabilitation services for those with severe injuries is supported by the current literature, which demonstrates improved outcomes for patients with severe TBI who complete specialized in-patient rehabilitation [5,55]. However, the above-mentioned findings are not entirely consistent with previous research [7,10,12] and should be interpreted with caution. It is worth mentioning that we used self-reports to assess the rehabilitation provision, which may have introduced a bias, as discussed in the limitation section. 

Overall, the present study highlights an inadequate provision of services, which leads to a high prevalence of unmet rehabilitation needs and emphasizes the necessity of more extensive and standardized assessment of functional impairments and corresponding rehabilitation needs. This finding may provide a starting point for further development of personalized and targeted interventions following TBI. 

### Strengths and Limitations of the Study

The present study’s strengths are its large sample size and the number of participating European countries, which together render a robust overview. Nevertheless, the study also exhibits some limitations. 

Firstly, patients with the two lowest functional recovery statuses were combined into one category, as the questionnaire version of the GOSE applied in the study cannot distinguish between vegetative status and severe disability. Further, functional impairments and the use of rehabilitation services were based on self-reports from the participants. By including the subjective experience of patients in a patient-centered rehabilitation, greater satisfaction, better adherence to treatment, and improved outcomes can be achieved; nevertheless, self-reported information can be biased [56], especially in those with impaired memory and self-awareness deficits [57]. To overcome this limitation, future studies, should combine self-reports with objective administrative data and clinical evaluations.

Secondly, overall, more than half of the initial group of patients did not provide information on different aspects of functioning and rehabilitation services. Among those were mostly male; they also had fewer years of education on average and resided predominantly in Western and Northern European countries. They were mostly admitted to the ICU, had significantly more severe injuries and suffered from more severe disabilities post-TBI. These findings suggest that non-participation could be related to an inability to complete self-report questionnaires due to cognitive impairments. Utilizing the perspective of the families or caregivers could allow for the inclusion of more severely injured patients. At the same time, however, it would likely introduce other biases such as the overestimation of problems—this may be related to a proxy’s inability to accurately assess a patient’s problems or the proxy’s own perceptions of what is important. 

Thirdly, to avoid losing statistical power, the study imputed missing predictor values using the MICE procedure. Nevertheless, the influence of the imputed values was negligible, since in the final model only injury severity (GCS) and brain injury severity (brain AIS) were retained. Both variables initially showed 3% and 1% missing values, respectively. Furthermore, even if a stepwise selection procedure for model building is sometimes criticized, backward selection appears to be the best of all stepwise approaches [43,58], and the bootstrapping validation prevented the drawing of premature conclusions. 

Finally, distinguishing between in- and out-patient rehabilitation was not possible in the regression model due to the relatively low number of observations. Therefore, future studies should provide a broader overview of various rehabilitation types by differentiating between in- and outpatient rehabilitation programs.

## 5. Conclusions

This study indicates numerous unmet rehabilitation needs across different outcome domains for individuals with moderate-to-severe disabilities after TBI. The study results emphasize the necessity of more extensive multidimensional and standardized assessments of functional and psychological impairments and the provision of corresponding rehabilitation services. Moreover, targeted rehabilitation programs aimed at improving outcomes should involve psychological services to meet the needs of individuals after TBI. Future research studies, which take into account objective administrative data, clinical evaluations, reports of caregivers and, as well as patients reports, will further improve knowledge about rehabilitation needs and services for TBI patients. 

## Figures and Tables

**Table 1:** Sample characteristics.

Variable	Group/Values	*N* (%) or *M* (*SD*) or *Md* [*Min*, *Max*]
Sex	Female	390 (32.3%)
	Male	816 (67.7%)
Age in years	*M* (*SD*)	49.3 (18.9)
	*Md* [*Min*, *Max*]	50.0 [16.00, 93.00]
	Missing	0 (0%)
Education in years	*M* (*SD*)	13.1 (3.84)
	*Md* [*Min*, *Max*]	13.0 [1.00, 30.00]
	Missing	321 (26.6%)
Employment status	Employed	633 (52.5%)
	Unemployed	104 (8.6%)
	Other	341 (28.3%)
	Missing	128 (10.6%)
Living status	Alone	948 (78.6%)
	Not alone	255 (21.1%)
	Missing	3 (0.2%)
Geographical regions	Western Europe	593 (49.2%)
	Northern Europe	306 (25.4%)
	Southern/Eastern Europe	307 (25.5%)
Premorbid somatic health status	Healthy	632 (52.4%)
	Mild disease	417 (34.6%)
	Severe disease	130 (10.8%)
	Missing	27 (2.2%)
Premorbid psychological problems	No	968 (80.3%)
	Yes	198 (16.4%)
	Missing	40 (3.3%)
Injury cause	Road traffic accident	550 (45.6%)
	Fall	455 (37.7%)
	Violent/other	196 (16.3%)
	Missing	5 (0.4%)
Clinical care pathways	ER	76 (6.3%)
	ward	271 (22.5%)
	ICU	859 (71.2%)
GOSE (6 months)	*M* (*SD*)	4.66 (1.17)
	*Md* [*Min*, *Max*]	5.00 [3.00, 6.00]
	Missing	0 (0%)
TBI severity	Mild	592 (49.1%)
	Moderate	141 (11.7%)
	Severe	425 (35.2%)
	Missing	48 (4.0%)
Abnormalities on the first CT scan	Absent	223 (18.5%)
	Present	750 (62.2%)
	Missing	233 (19.3%)
Brain Injury AIS	*M* (*SD*)	3.82 (1.25)
	*Md* [*Min*, *Max*]	4.00 [1.00, 6.00]
	Missing	19 (1.6%)
Total ISS	*M* (*SD*)	27.7 (16.2)
	*Md* [*Min*, *Max*]	25.0 [1.00, 75.0]
	Missing	19 (1.6%)
Total		1206 (100%)

Note. Percentages may contain rounding errors in the second decimal place. *M* = mean; *SD* = Standard deviation; *Md* = median; *Min* = minimum; *Max* = maximum; Employment status = employed (full-time employed, part-time employed, on sick leave, special/sheltered employment), unemployed (looking for work, unemployed, unable to work), other (retired, student/school-going, homemaker); Geographical regions = Western Europe (Austria, Belgium, France, Germany, the Netherlands, United Kingdom); Northern Europe (Denmark, Finland, Latvia, Lithuania, Norway, Sweden); Southern/Eastern Europe (Italy, Spain, Hungary, Romania, Serbia); Premorbid somatic health status is based on the ASA-PS classification; ER = emergency room; ward = admission to hospital ward; ICU = intensive care unit; CT = computed tomography; AIS = Abbreviated Injury Scale; ISS = Injury Severity Score.

**Table 2:** Impairment in the different outcome areas at three and six months after TBI.

Outcome Type	Impairment	Three Months	Six Months	*p*
		*N* (%)	*N* (%)	
Problems with activities of daily life	Not impaired	283 (23.5%)	361 (29.9%)	0.01
	Impaired	411 (34.1%)	400 (33.2%)	
	Missing	512 (42.5%)	445 (36.9%)	
Physical problems	Not impaired	289 (24.0%)	333 (27.6%)	0.06
	Impaired	506 (42.0%)	480 (39.8%)	
	Missing	411 (34.1%)	393 (32.6%)	
Cognition problems	Not impaired	209 (17.3%)	215 (17.8%)	0.41
	Impaired	498 (41.3%)	567 (47.0%)	
	Missing	499 (41.4%)	424 (35.2%)	
Speech and language problems	Not impaired	579 (48.0%)	607 (50.3%)	0.49
	Impaired	215 (17.8%)	207 (17.2%)	
	Missing	412 (34.2%)	392 (32.5%)	
Psychological problems	Not impaired	411 (34.1%)	454 (37.6%)	0.70
	Impaired	277 (23.0%)	292 (24.2%)	
	Missing	518 (43.0%)	460 (38.1%)	

Note. Percentages may contain rounding errors in the second decimal place. *N* = number of observations; % = percentage; Problems with activities of daily life (QOLIBRI scale Daily Life and Autonomy, score > 60); Physical problems (problems with mobility/walking, problems with moving arms and hands); Cognition problems (RPQ: ‘Forgetfulness, poor memory’ or ‘Poor concentration’, or ‘Taking longer to think’; score ≥ 2); Speech/language problems (problems with speaking or understanding others); Psychological problems (one of the following outcomes considered impaired according to the respective cut-off scores: PTSD: PCL-5 score ≥ 33; Depression: PHQ-9 score ≥ 10; Anxiety: GAD-7 score ≥ 10); *p = p*-values are obtained from the chi-square tests and are significant at <1% (Bonferroni-adjusted alpha level for multiple comparisons: 0.05/5).

**Table 3:** Professional help provided for patients with impaired outcomes at three and six months.

Outcome Type	Type of Professional Help	Three Months			Six Months			*p*
		Impaired	Help Provided	%	Impaired	Help Provided	%	
Problems with activities of daily life	Occupational therapy	411	153	37%	400	170	43%	0.34
Physical problems	Physiotherapy	506	334	66%	480	340	71%	0.82
Cognition problems	Cognitive rehabilitation	498	166	33%	567	212	37%	0.02
Speech and language problems	Speech therapy	215	84	39%	207	89	43%	0.70
Psychological problems	Psychological services	277	73	26%	292	89	30%	0.21

Note. Impaired = number of individuals considered impaired according to the respective cut-offs; Help provided = number of individuals received respective rehabilitation services based on self-report; % = relative frequency of individuals received respective rehabilitation services; *p* = *p*-values are obtained from the chi-square tests comparing distributions of provided professional help between three and six months and are significant at <1% (Bonferroni-adjusted alpha level for multiple comparisons: 0.05/5).

**Table 4:** Rehabilitation services received at there and six months (total and split by recovery status at six months).

Recovery Status	Rehabilitation	Three Months	Six Months	*p*
		*N* (%)	*N* (%)	
Total	No	306 (25.4%)	276 (22.9%)	0.07 ^‡^
	In-patient	341 (28.3%)	356 (29.5%)	
	Out-patient	150 (12.4%)	183 (15.2%)	
	Missing	409 (33.9%)	391 (32.4%)	
	Total	1206 (100.0%)	1206 (100.0%)	
GOSE 2/3 (vegetative state/lower severe disability)	No	30 (9.7%)	18 (5.8%)	0.05 ^†^
	In-patient	103 (33.3%)	93 (30.1%)	
	Out-patient	9 (2.9%)	18 (5.8%)	
	Missing	167 (54.0%)	180 (58.3%)	
	Total	309 (100.0%)	309 (100.0%)	
GOSE 4 (upper severe disability)	No	35 (20.1%)	33 (19.0%)	0.34 ^†^
	In-patient	51 (29.3%)	68 (39.1%)	
	Out-patient	14 (8.0%)	23 (13.2%)	
	Missing	74 (42.5%)	50 (28.7%)	
	Total	174 (100.0%)	174 (100.0%)	
GOSE 5 (lower moderate disability)	No	95 (28.0%)	75 (22.1%)	0.12 ^†^
	In-patient	100 (29.5%)	98 (28.9%)	
	Out-patient	52 (15.3%)	67 (19.8%)	
	Missing	92 (27.1%)	99 (29.2%)	
	Total	339 (100.0%)	339 (100.0%)	
GOSE 6 (upper moderate disability)	No	146 (38.0%)	150 (39.1%)	0.89 ^†^
	In-patient	87 (22.7%)	97 (25.3%)	
	Out-patient	75 (19.5%)	75 (19.5%)	
	Missing	76 (19.8%)	62 (16.1%)	
	Total	384 (100.0%)	384 (100.0%)	

Note. Percentages may contain rounding errors in the second decimal place. *N* = number of cases, % = percentage *p* = *p*-values are obtained from the chi-square tests comparing distributions of provided rehabilitation services at three and six months and are significant at ^‡^ <5% and at ^†^ <1.25% (Bonferroni-adjusted alpha level for multiple comparisons: 0.05/4).

**Table 5:** Rehabilitation provided to patients with impaired outcomes.

Imapired Outcome	Rehabilitation ^‡^	Three Months	Six Months	*p*
Problems with activities of daily life	No	153 (37.2%)	116 (29.0%)	0.03
	In-patient	164 (39.9%)	178 (44.5%)	
	Outpatient	82 (20.0%)	98 (24.5%)	
	Missing	12 (2.9%)	8 (2.0%)	
Physical problems	No	175 (34.6%)	145 (30.2%)	0.06
	In-patient	240 (47.4%)	223 (46.5%)	
	Outpatient	87 (17.2%)	110 (22.9%)	
	Missing	4 (0.8%)	2 (0.4%)	
Cognition problems	No	182 (36.5%)	170 (30.0%)	0.05
	In-patient	200 (40.2%)	236 (41.6%)	
	Outpatient	105 (21.1%)	146 (25.7%)	
	Missing	11 (2.2%)	15 (2.6%)	
Speech and language problems	No	62 (28.8%)	51 (24.6%)	0.06
	In-patient	118 (54.9%)	104 (50.2%)	
	Outpatient	34 (15.8%)	52 (25.1%)	
	Missing	1 (0.5%)	0 (0.0%)	
Psychological problems	No	123 (44.4%)	108 (37.0%)	0.19
	In-patient	96 (34.7%)	109 (37.3%)	
	Outpatient	53 (19.1%)	68 (23.3%)	
	Missing	5 (1.8%)	7 (2.4%)	

^‡^ Note that the type of rehabilitation is independent of the impaired outcome. Note. Percentages may contain rounding errors in the second decimal place. *p* = *p*-values are obtained from the chi-square tests comparing distributions of provided rehabilitation services at 3 and 6 months and are significant at <1% (Bonferroni-adjusted alpha level for multiple comparisons: 0.05/5).

**Table 6:** Results of the logistic regression (final model).

Variable/Category	Reference Group	Estimate	*S.E.*	*p*	*OR*	CI 2.5%	CI 97.5%
Intercept	-	1.38	0.80	0.086	3.99	0.82	19.29
GCS	-	−0.24	0.04	**<0.001**	0.79 ^‡^	0.72	0.86
Brain Injury AIS	-	0.56	0.16	**0.001**	1.75 ^☥^	1.27	2.42
ISS	-	−0.02	0.01	0.063	0.98	0.96	1.00
Physical problems	-	0.65	0.24	**0.006**	1.92 ^☥^	1.21	3.05
Cognition	-	1.39	0.27	**<0.001**	4.00 ^☥^	2.34	6.83
Psychological problems	-	−0.56	0.25	**0.026**	0.57 ^‡^	0.35	0.93
Ward	ER	−0.86	0.44	0.052	0.42	0.18	1.00
ICU	ER	−0.38	0.55	0.499	0.69	0.23	2.04

^‡^ Lower probability of receiving rehabilitation services; ^☥^ higher probability of receiving rehabilitation services; Note. Estimate = regression coefficient; *S.E.* = standard error; *p* = *p*-value; *OR* = odds ratio; CI 2.5% = lower bound of the 95% confidence interval; CI 97.5% = upper bound of the 95% confidence interval; bold *p*-values are significant at 5% α-level; GCS = Glasgow Coma Scale; Brain Injury AIS = Abbreviated Injury Scale, Brain Injury severity; ISS = total Injury Severity Score; ER = emergency room; ward = admission at hospital ward; ICU = intensive care unit.

## Data Availability

All relevant data are available upon request from CENTER-TBI, and the authors are not legally allowed to share it publicly. The authors confirm that they received no special access privileges to the data. CENTER-TBI is committed to data sharing and in particular to responsible further use of the data. Hereto, we have a data sharing statement in place: https://www.center-tbi.eu/data/sharing. The CENTER-TBI Management Committee, in collaboration with the General Assembly, established the Data Sharing policy, and Publication and Authorship Guidelines to assure correct and appropriate use of the data as the dataset is hugely complex and requires help of experts from the Data Curation Team or Bio- Statistical Team for correct use. This means that we encourage researchers to contact the CENTER-TBI team for any research plans and the Data Curation Team for any help in appropriate use of the data, including sharing of scripts. Requests for data access can be submitted online: https://www.center-tbi.eu/data. The complete Manual for data access is also available online: https://www.center-tbi.eu/files/SOP-Manual-DAPR-20181101.pdf.

## Supplementary Materials

The following are available online at https://www.mdpi.com/2077-0383/10/5/1035/s1: list of CENTER-TBI participants and investigators.

## Appendix A

The Appendix A contains additional tables.

**Table A1:** In- and out-patient rehabilitation provided at 3 and 6 months after TBI by clinical pathways.

Rehabilitation	3 Months				6 Months				*p*
	ER	Ward	ICU	Total	ER	Ward	ICU	Total	
No	43 (56.6%)	126 (46.5%)	137 (15.9%)	306 (25.4%)	36 (47.4%)	125 (46.1%)	115 (13.4%)	276 (22.9%)	0.61
In-patient	2 (2.6%)	36 (13.3%)	303 (35.3%)	341 (28.3%)	4 (5.3%)	21 (7.7%)	331 (38.5%)	356 (29.5%)	0.06
Out-patient	13 (17.1%)	38 (14.0%)	99 (11.5%)	150 (12.4%)	15 (19.7%)	48 (17.7%)	120 (14.0%)	183 (15.2%)	0.98
Missing	18 (23.7%)	71 (26.2%)	320 (37.3%)	409 (33.9%)	21 (27.6%)	77 (28.4%)	293 (34.1%)	391 (32.4%)	
Total	76 (6.3%)	271 (22.5%)	859 (71.2%)	1206 (100%)	76 (6.3%)	271 (22.5%)	859 (71.2%)	1206 (100%)	

Note. Percentages may contain rounding errors in the second decimal place. ER = emergency room; ward = hospital ward; ICU = intensive care unit; *p* = *p*-values are obtained from a two-dimensional chi-square test comparing distributions of the clinical care pathways in rehabilitation groups at three and six months and are significant at <1.7% (Bonferroni-adjusted alpha level for multiple comparisons: 0.05/3).
